# PCR vs karyotype for CVS and amniocentesis—the experience at one tertiary fetal medicine unit

**DOI:** 10.1007/s11845-021-02715-y

**Published:** 2021-07-20

**Authors:** Catherine Finnegan, Suzanne Smyth, Orla Smith, Karen Flood, Jane Dalrymple, Fionnuala M. Breathnach, Fergal D. Malone

**Affiliations:** 1 Department of Obstetrics and Gynaecology, Royal College of Surgeons in Ireland, Rotunda Hospital, Parnell Square, Dublin 1, Ireland; 2 Fetal Medicine Department, Rotunda Hospital, Parnell Square, Dublin 1, Ireland

**Keywords:** CVS, Amniocentesis, Karyotype, PCR

## Abstract

**Purpose:**

Despite the rise of non-invasive screening tests for fetal aneuploidy, invasive testing during pregnancy remains the definitive diagnostic tool for fetal genetic anomalies. Results are rapidly available with polymerase chain reaction (PCR) tests, but cases have been reported whereby initial results were not confirmed after pregnancy termination and the fetal karyotype was ultimately normal. We sought to examine the potential discordance between PCR and karyotype for fetal aneuploidy.

**Methods:**

The results from all amniocentesis and CVS tests performed over a 6-year period in a large tertiary level fetal medicine unit were reviewed. The results of PCR and karyotype were recorded and discrepancies examined. Pregnancy outcomes were also recorded.

**Results:**

A total of 1222 invasive tests were performed (716 amniocentesis and 506 CVS). Within the cohort having amniocentesis, 11 had discrepant results (normal QF-PCR result but with a subsequent abnormal karyotype). There was 1 case among this group which QF-PCR should have identified. Within the CVS group, 7 patients had discrepant results. All had a diploid QF-PCR and would not have been identified as abnormal by it.

**Conclusion:**

PCR can be reliably used to determine aneuploidy of chromosomes 13, 18, and 21. However, in cases of sex chromosome aneuploidy, its performance is less reliable and warrants waiting for a complete karyotype. Given such discordance, we advise waiting for karyotype for all invasive tests performed in the presence of a normal ultrasound before advising a patient of a diploid QF-PCR result or potentially terminating a normal pregnancy.

## Introduction

It is estimated that up to 5% of the pregnant population in the UK will be offered invasive testing [1]. This may be as a result of a high-risk screening test, a fetal structural anomaly found on ultrasound, or a previous history. The selection of chorionic villus sampling (CVS) or amniocentesis depends on the gestational age such that CVS is performed between 11 + 0 and 13 + 6 weeks of gestation and involves aspiration of placental villi. It can be done via the transabdominal or transcervical route, depending on the location of the placenta. Amniocentesis is performed from 15 weeks of gestation onwards following the fusion of the chorion and amnion. The disadvantage of these tests is that miscarriage may occur. A large randomized trial of 4606 low-risk women quoted this risk to be 1% [2].

Non-invasive prenatal testing (NiPT) has become increasingly popular worldwide, and the market for it is growing annually. The basis for its popularity lies in the absence of risk of miscarriage and the early gestational age for screening results. However, despite the advantages of NIPT, it is still only a screening test and an invasive test must be performed in order to make a definitive diagnosis of fetal aneuploidy.

When an invasive test is performed, there are options for rapid genetic testing with quantitative fluorescent polymerase chain reaction (QF-PCR) or fluorescent in situ hybridization (FISH) tests. This can take 2–3 days for evaluation of chromosomes 13, 18, and 21 and the sex chromosomes. Alternatively, a full karyotype can be performed which may require 2–3 weeks to obtain a result.

We sought to examine the performance of QF-PCR in comparison to karyotype in our institution for both CVS and amniocentesis.

## Methods

This was a retrospective review of all amniocentesis and CVS procedures performed at a large tertiary level fetal medicine unit over a 6-year period from January 2013 until December 2018. The cases were found using the hospital annual clinical reports and cross-checked within the Fetal Medicine Department using the Viewpoint™ software program. For each of these cases, the type of test and results of the genetic testing were noted. From this, the results of QF-PCR and karyotype were compared for discordance.

## Results

A total of 1222 invasive tests were performed (716 amniocentesis and 506 CVS). Within the cohort having amniocentesis, 11 had a normal PCR result but with a subsequent abnormal karyotype. Table 1 shows the results, with “diploid” representing a normal complement of chromosomes 13, 18, and 21 and the sex chromosomes.

**Table 1:** Discordance between PCR and karyotype on amniocentesis (*n* = 716)

QF-PCR	Karyotype	Outcome
Diploid	46XY, deletion chromosome 8	TOF, pulmonary hypoplasia, SVD 40 weeks
Diploid	46XX, deletion 22q	TOP
Diploid	46XY, duplication chromosome 10	IUGR, VSD, 36/40 SVD
Diploid	47XY + 2	TOP
Diploid	47XY + 12	TOP
Diploid	47XY + 12	TOP
Diploid	46XY inverted chromosome 7	LSCS 4.4 kg, baby well
Diploid	47XY + r[23]/46XY[17]ish	TOP
Diploid	46XX del 22q (Phelan-McDermid syndrome)	IUD
Diploid	46XX ring chromosome 13	TOP
Diploid	45X	19/40 miscarriage

There was one case among this group that PCR should have identified. The case was an amniocentesis performed following diagnosis of cystic hygroma in a fetus noted also to be small for gestational age. The PCR was normal, but the karyotype was 45X. The pregnancy ended in miscarriage at 19 weeks of gestation.

Within this group, there was one amniocentesis performed for multiple anomalies on ultrasound with a QF-PCR result showing T13; however, the karyotype result was 46XY. An intrauterine death was confirmed at 35/40 and diagnosis confirmed on postmortem.

Therefore, within our institution, when amniocentesis was performed, in 99.9% (715/716) of cases the QF-PCR was correct.

Within the CVS group, seven patients had discrepant results. All had a diploid QF-PCR result. Table 2 displays the discordant CVS results.

**Table 2:** Discordance between PCR and karyotype on CVS (*n* = 506)

QF-PCR	Karyotype	Outcome
Diploid	46XY copy number loss chromosome 13	TOP
Diploid	46XX diGeorge syndrome	TOP
Diploid	47XY + 7[39]/46XX[11]	Placental mosaicism
Diploid	3 cell lines	Normal amniocentesis—mosaicism
Diploid	47XY + 12	TOP
Diploid	47XY + 2	TOP
Diploid	46X-translocation X–Y	TOP

An interesting case among this group was a patient referred for CVS on a background of non-invasive screening which revealed a high-risk result for Turner syndrome. The PCR was normal, but karyotype ultimately revealed 46X with an unbalanced translocation between the short arm of chromosome X and the long arm of Y. Of note, this patient had no anomalies identified on ultrasound. The pregnancy ended by termination.

When we looked at this more closely with the genetics laboratory, they stated that a combination of marker location and the limited number of informative markers for the QF-PCR were suggestive but not conclusive of a sex chromosome rearrangement. In view of this, they reported it as an uninformative result for the sex chromosomes by QF-PCR and recommended waiting for karyotype to clarify the sex chromosome results. The 46,X,der(Y)t(X;Y)(p22.31;q11.221) caused the following:

1. An 8.36-Mb duplication of the short arm of chromosome X within bands p22.33 to p22.31.
  There are two QF-PCR markers that suggest there may be additional X material present:
  X10 at Xp22.32 had a 1:1 ratio, indicating there was additional X chromosome material at that location. XY3 at Xp22.33/Yp11.32 had a 2:1 ratio, indicating there was additional material present on either the X or Y at that location. However, XY3 is known to have benign copy number variations that can affect the ratio.
  AMELXY had a normal ratio, indicating an equal amount of X and Y (this is the next marker along).
2. A 43.1-Mb deletion of the long arm of chromosome Y within bands q11.221 to q12. There are no QF-PCR markers covering this deleted Y region.
  Within the 506 CVS performed, one case with QF-PCR did not identify a sex chromosome abnormality which was subsequently detected with karyotype. One hundred percent (506/506) of cases were correctly identified by QF-PCR.
  When we look at the abnormal QF-PCR results in this group and compare them to karyotype, one CVSn performed for a large cystic hygroma gave a QF-PCR result of T21, but karyotype returned 46XX. The pregnancy ended in miscarriage at 14 weeks of gestation.

Therefore, in our institution, when all invasive tests are examined, in 99.9% (1221/1222) of cases the QF-PCR correctly identified those abnormalities it should have.

## Discussion

QF-PCR is based on the amplification of chromosome-specific DNA sequences (STR, short tandem repeats) polymorphic in length between subjects. By means of fluorescent primers, the amplified segments can be visualized and quantified as peak areas on automated DNA scanners. Heterozygous subjects are expected to show two peaks (a peaks ratio of 1:1) for each chromosome analyzed, while trisomies for example are seen either as an extra peak (triallelic subjects) or as a 2:1 ratio peak between the two areas [3].

With abnormalities of chromosomes 13, 18, and 21 and the sex chromosomes being the most common aneuploidies [4], rapid QF-PCR testing will be performed in the majority of cases. However, there have been cases reported, with some reaching the media, whereby QF-PCR was reported as abnormal, the pregnancy was terminated, and the karyotype later returned a normal result. In some rare instances, this may have been due to placental mosaicism when a CVS was performed, but in the case of amniocentesis, it illustrates a false-negative result from the test. Waiting for a full karyotype to be resulted carries its own disadvantages. If a karyotype is requested, the delay in obtaining a result of 2–3 weeks places the added burden and psychological distress on a patient and their family of not only waiting longer for a result, but also that if a termination of pregnancy is their chosen path, it must now be performed at a later gestation.

Several other groups have previously looked at how QF-PCR testing performs in comparison to conventional karyotype. In 2001, Levett et al. compared amniocentesis QF-PCR results to karyotype in over 5000 patients. They found that QF-PCR from amniocentesis correctly identified 100% of cases of trisomy 13, 18, and 21 and triploidy [4]. However, they did explore how different markers on sex chromosomes can vary the results and therefore recommended in future that these be employed. A group in Brazil sought to examine the efficiency of QF-PCR in comparison to conventional cytogenetics as it was not being offered to public healthcare patients. They found that QF-PCR correctly identified and agreed with karyotype results in 98% of their samples. Missed were either mosaic or resulted from chromosomal rearrangement, and thus they recommend these still undergo conventional karyotyping [5]. This study was limited in that they only examined 162 samples. Similar results are found throughout the literature. Lildballe et al. analyzed 2550 samples from chorionic villus sampling (CVS) and amniotic fluid from high-risk pregnancies and reported positive and negative predictive values greater than 99.8% [6]. Łaczmańska et al. [7] analyzed 100 samples of amniotic fluid and reported concordance in 95 cases (95%). Rostami et al. [8] reported 4058 samples analyzed for QF-PCR with a concordance of 98.59%. Tekcan et al. [9] compared 100 amniotic fluid samples with karyotype results and obtained 99% concordance on 100 samples, including 4 abnormalities. More recently, Sun et al. also found QF-PCR was comparable to conventional cytogenetic testing [10]. However, in all of these studies, it is worth noting that small numbers of genetic anomalies were detected overall, and this should be kept in mind when reviewing predictive values.

We have found that QF-PCR can be reliably used to determine aneuploidy of chromosomes 13, 18, and 21. In those cases in which QF-PCR did not identify aneuploidy (the case of ring chromosome 13), there were multiple abnormal features on ultrasound. In relation to the cases of sex chromosome abnormality (a case of 45X and a case of unbalanced translocation between the short arm of chromosome X and the long arm of Y), the performance of QF-PCR is less reliable and may warrant waiting for a complete karyotype. In the case of QF-PCR showing aneuploidy, but karyotype not, both of these cases had ultrasound abnormalities.

Therefore, given the minimal risk of discordance, we advise waiting for karyotype for invasive tests performed in the event of no abnormal ultrasound features before advising a patient of reassuring test results.
